# A Case of Carcinoma of the Cervix Uteri

**Published:** 1886-07-20

**Authors:** Virgil O. Hardon

**Affiliations:** Lecturer upon Operative Gynæcology in the Southern Medical College, Atlanta, Ga.


### A CASE OF CARCINOMA OF THE CERVIX UTERI.

By VIRGIL O. HARDON, M. D.,
Lecturer upon Operative Gynæcology in the Southern Medical College, Atlanta, Ga.

In the *Nouvelles Archives d' Obstetrique et de Gynecologie* of March 25, 1886, there appeared an article by Madame Gaches Sarraute, entitled "The Palliative Treatment of Cases of Cancer of the Womb which do not admit of Operation." In this article the authoress describes a plan of treatment which was employed in twenty-five cases in the service of Siredy at the Lariboisiere Hospital in Paris. This treatment consisted of copious antiseptic injections by means of a fountain syringe, continued at each sitting until all the *debris* of necrosed and broken-down tissue was removed from the ulcerated surface. This was followed by the application of a tampon of cotton saturated with a 4-per cent. solution of hydrate of chloral, and thickly covered with iodoform. This tampon was of such size as to completely cover the ulcerated surface. This treatment was employed three times a week. The results are summarized by Madame Sarraute as follows:

"1. If the dressings are applied regularly, they completely check the hemorrhage, no matter how profuse it may be.

"2. They change the nature of the ulcerated surface, cleansing it of the putrilaginous matter which forms upon it, and giving to it the appearance of a healthy sore.

"3. At the beginning, they quickly relieve the pains. These reappear with great intensity, but, finally, after a variable time, they cease altogether.

"4. They have great influence upon the general condition of the patient, and prevent auto-infection by resorption of the infectious matter which forms upon the diseased surface.

"5. They limit the extension of the lesion, and prevent neighboring organs from becoming involved.

"6. They permit the patient to live a comfortable and useful life, and to conceal, so to speak, her disease."

Soon after reading this article I chanced to be called to a case of this kind, and I employed the treatment described above, with what result will appear from the following report of the case :

R. F——, married, aged fifty-three, a robust, hard-working woman of Welch birth ; has borne five children, and had four miscarriages which she attributes to hard work ; has always enjoyed perfect health No uterine symptoms until about a year ago, when her menses, which had continued with regularity up to that time, became very profuse, and lasted two weeks of each month. This continued for five months, and, since that time, she has had hemorrhages at irregular intervals. When the menses became irregular she began to suffer severe pains in the hypogastrium, which increased in severity until she was obliged to take to the bed and use opiates. In the intervals of hemorrhages a watery discharge from the vagina was present. She rapidly lost flesh, strength, and appetite. She was treated by various practitioners, regular and irregular, one of whom amputated a portion of the cervix uteri. Aside from this, her treatment, as far as can be learned, consisted principally of opiates.

When first seen by me—April 27—she had been in bed eight months ; was greatly emaciated ; unable to retain any food, and suffered from pains which necessitated the constant use of opiates ; bowels were constipated, micturition frequent and painful. Examination showed the cervix to have been entirely destroyed, and its stump presented a broken-down, suppurating surface which bled at the slightest touch. Above this necrosed portion the tissues were indurated, and the womb was immovably fixed. The induration extended into the surrounding pelvic cellular tissue, and involved the vesico-vaginal and recto-vaginal septa. The ulceration was limited to the cervix. There was marked fetor and constant serous discharge, mixed with sanious pus.

The treatment described by Sarraute was applied every second day, carbolic acid being used as a disinfectant. The use of opiates was discontinued after the second day, and powders of pepsin and bismuth were given every three hours. The bowels were kept open by laxatives.

At the end of a week the patient was *absolutely* free from pain, the fetor and discharge had ceased, and the ulcerated surface had the appearance of a healthy, granulating sore ; appetite had returned, and the stomach retained food. During the second week she sat up an hour or two every day, and began to gain flesh and strength. On the nineteenth day of treatment, and every day following, she went out on the piazza and sat up nearly all day. On the twenty-sixth day she went out in the garden and gathered a large boquet, which she presented to me when I made my visit. Her improvement was uninterrupted. and no hemorrhage occurred while she was under my treatment. On the twenty-eighth day there was slight return of pain, but not severe enough to require the use of an anodyne.

At this time she was persuaded by a neighbor to place herself in the hands of a female charlatan, who promised to cure her, a promise which, of course, I could not make. She, therefore, passed from under my observation ; but I have learned that she began to decline from that time, all her old symptoms returned, and she died with symptoms of septic poisoning in less than a month.

The improvement in this case was so rapid and uninterrupted that I am convinced that the treatment of Sarraute is a decided advance upon any method heretofore in vogue. I report this case that others may be induced to give this method a trial, and report the results obtained.

